# The deubiquitylase UCHL3 maintains cancer stem-like properties by stabilizing the aryl hydrocarbon receptor

**DOI:** 10.1038/s41392-020-0181-3

**Published:** 2020-06-17

**Authors:** Lianlian Ouyang, Bin Yan, Yating Liu, Chao Mao, Min Wang, Na Liu, Zuli Wang, Shouping Liu, Ying Shi, Ling Chen, Xiang Wang, Yan Cheng, Ya Cao, Desheng Xiao, Lingqiang Zhang, Shuang Liu, Yongguang Tao

**Affiliations:** 1 Department of Oncology, Institute of Medical Sciences, National Clinical Research Center for Geriatric Disorders, Xiangya Hospital, Central South University, 410008 Changsha, Hunan China; 2 Key Laboratory of Carcinogenesis and Cancer Invasion, Ministry of Education, Department of Pathology, Xiangya Hospital, Central South University, 410078 Changsha, Hunan China; 3 Key Laboratory of Carcinogenesis of the Ministry of Health, Cancer Research Institute; School of Basic Medicine, Central South University, 410078 Changsha, Hunan China; 4 Department of Thoracic Surgery, Hunan Key Laboratory of Tumor Models and Individualized Medicine, Second Xiangya Hospital, Central South University, Changsha, China; 5 Xiangya School of Pharmaceutical Sciences, Central South University, 410008 Changsha, China; 6 Department of Pathology, Xiangya Hospital, Central South University, 410008 Changsha, Hunan China; 7 State Key Laboratory of Proteomics, National Center for Protein Sciences (Beijing), Beijing Institute of Lifeomics, 100850 Beijing, China

**Keywords:** Cancer stem cells, Lung cancer

## Abstract

Cancer stem cells (CSCs) exhibit highly aggressive and metastatic features and resistance to chemotherapy and radiotherapy. Aryl hydrocarbon receptor (AhR) expression varies among non-small cell lung cancers (NSCLCs), and the mechanisms that support abnormal AhR expression in CSCs remain elusive. Here, we identified ubiquitin carboxyl terminal hydrolase L3 (UCHL3), a DUB enzyme in the UCH protease family, as a bona fide deubiquitylase of the AhR in NSCLC. UCHL3 was shown to interact with, deubiquitylate, and stabilize AhR in a manner dependent on its deubiquitylation activity. Moreover, we showed that UCHL3 promotes the stem-like characteristics and potent tumorigenic capacity of NSCLC cells. UCHL3 increased AhR stability and the binding of AhR to the promoter regions of the “stemness” genes ATP-binding cassette subfamily G member 2 (ABCG2), KLF4, and c-Myc. Depletion of UCHL3 markedly downregulated the “stemness” genes ABCG2, KLF4, and c-Myc, leading to the loss of self-renewal and tumorigenesis in NSCLCs. Furthermore, the UCHL3 inhibitor TCID induced AhR degradation and exhibited significantly attenuated efficacy in NSCLC cells with stem cell-like properties. Additionally, UCHL3 was shown to indicate poor prognosis in patients with lung adenocarcinoma. In general, our results reveal that the UCHL3 deubiquitylase is pivotal for AhR protein stability and a potential target for NSCLC-targeted therapy.

## Introduction

Proteins are decorated with a diverse array of posttranslational modifications (PTMs) that regulate their spatial and temporal functions. Protein ubiquitination is a posttranslational modification that regulates all kinds of biological processes by influencing the stabilization, localization and function of substrate proteins.^1^ Ubiquitination, a highly regulated posttranslational protein modification,^2^ is reversible by reactions catalyzed by several distinct families of deubiquitylases.^3^ Deubiquitinating enzymes (DUBs), which can remove ubiquitin from protein substrates, protect proteins from degradation, following which free ubiquitin is released to participate in the cyclic ubiquitination reaction. Nevertheless, in some cases, DUBs can also promote substrate degradation.^4,5^ The balance between ubiquitination and deubiquitination is indispensable for all kinds of biological processes.^6,7^

The DUB enzymes identified are divided into five subfamilies,^8–11^ one of which is the ubiquitin C-terminal hydrolase (UCH) family. Four UCH family members have been identified: UCHL1, UCHL3, UCH37 and BRCA1-associated protein-1 (BAP1),^12–14^ and all UCH enzymes possess a conserved catalytic domain (UCH domain) composed of 230 amino acids.^7^ As the homology between UCHL3 and UCHL1 is as high as 53%, they are the closest family members, but UCHL3 and UCHL1 have very different biochemical characteristics.^15^

Because of its deneddylation activity, UCHL3 appears to be unique in the UCH family.^16^ Some research has suggested that UCHL3 plays a role in tumorigenesis and that UCHL3 expression is upregulated in breast cancer and cervical cancer tissues.^17,18^ However, the specific mechanism and role of UCHL3 in tumorigenesis have not been clarified.

Aryl hydrocarbon receptor (AhR) belongs to the basic helix-loop/PER-ARNT-SIM (bHLH-PAS) transcription factor family, the members of which require ligand activation. Its classical ligand, TCDD (2,3,7,8-tetrachlorodibenzo-p-dioxin), is widespread in industrial environmental pollutants (in the atmosphere, food and water sources) and associated with severe hepatotoxicity and skin toxicity.^19,20^ AhR expression in lung cancer is complicated. Some reports indicate that AhR is downregulated in lung cancer,^21^ whereas others report that AhR is overexpressed.^22,23^ AhR in the cytoplasm is in a resting state, and after its activation, AhR binds its nuclear transporter, ARNT, to form an AhR-ARNT heterodimer that enters the nucleus, where it initiates the transcription of its target genes.^20^ We recently found that benzopyrene (BaP) promotes nuclear transport by activating AhR, leading to malignant transformation of NSCLC.^24^ Our previous studies also found that AhR activates downstream target genes in a ligand-independent manner.^25^ In addition, activation of the AhR signaling pathway was shown to be related to radiation resistance and the stem-like characteristics of cancer cells, whereas AhR knockout reduced the stem-like phenotype of cancer cells.^26^

Cancer stem cells (CSCs), a small cell population in cancer tissues with stem cell characteristics, have the ability to undergo self-renewal and the potential for nondirectional differentiation; they can differentiate into different types of cancer cells with different degrees of differentiation.^27,28^ Stem cell characteristics have become a target of cancer therapy.^27,29–32^ Researchers have identified markers of cancer stem cells, such as CD44, CD133, ATP binding cassette transporter G2 (ABCG2), aldehyde dehydrogenase 1 (ALDH1), KLF4, Oct4, c-Myc, and Nanog,^33–36^ which are useful to diagnose the degree of CSC malignancy.

Among all cancers, lung cancer accounts for the most deaths, and lung cancer is the most common cancer in China and the world.^37^ Lung cancer can be divided into small cell lung cancer and non-small cell lung cancer (NSCLC), and NSCLC can be subdivided into adenocarcinomas (ADC) and squamous cell carcinoma (SCC), which account for 80–85% of all lung cancer cases.^38^

In this study, we found that UCHL3 is a contributing factor to cancer stem-like properties that promotes tumorigenesis by stabilizing AhR protein degradation.

## Results

### UCHL3 is upregulated in NSCLC and associated with poor prognosis in lung ADC

**Fig. 1:**
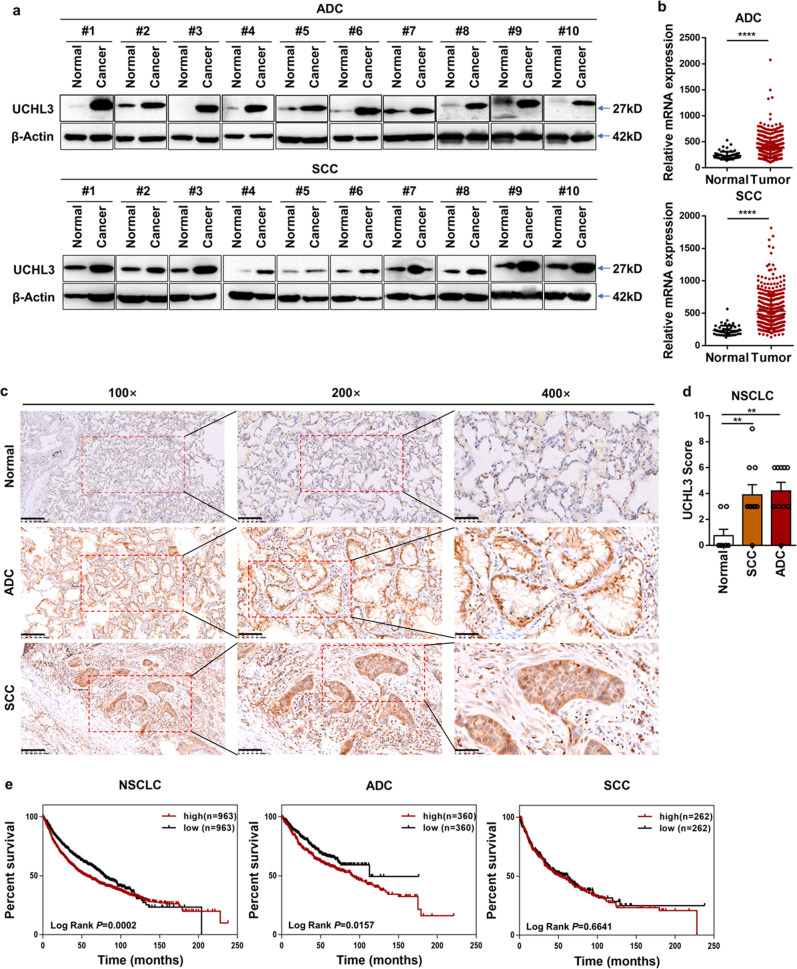
UCHL3 is upregulated and associated with poor survival in NSCLC. **a** Western blot analysis showed increased UCHL3 protein expression in 20 paired lung cancer (ADC and SCC) tissue samples relative to adjacent normal lung tissue samples. **b** TCGA analysis of UCHL3 mRNA expression in ADC (Normal *n* = 58; Tumor *n* = 488) and SCC (Normal *n* = 50; Tumor *n* = 409) lung cancer samples compared to normal lung samples. Each dot represents a sample. Data are shown as the mean ± SD; ****p < 0.0001. **c** IHC analysis showed increased UCHL3 expression levels in lung cancer (magnification, ×100 scale bar = 200 μm; magnification, ×200 scale bar = 100 μm; magnification, ×400 scale bar = 50 μm). **d** IHC scores indicating UCHL3 expression levels in lung cancer tissues versus normal tissues (Normal *n* = 8; SCC *n* = 10; ADC *n* = 10). Data are shown as the mean ± SD; **p < 0.01. **e** Kaplan–Meier curves showing overall survival rates associated with UCHL3 expression in lung cancer. NSCLC *p* = 0.0002; ADC *p* = 0.0157; SCC *p* = 0.6641; tested by log-rank test. Two-tailed Student’s *t*-test (**b**) or one-way ANOVA with multiple comparisons (**d**)

To clarify the role of UCHL3 in lung cancer, we used Western blotting to detect the expression of UCHL3 in 20 pairs of NSCLC tissues and paracancerous normal tissues. Upregulation of UCHL3 was observed in both ADC and SCC (Fig. 1a). In addition, TCGA database analysis showed that UCHL3 mRNA levels were upregulated in both ADC and SCC (Fig. 1b). We carried out immunohistochemical analysis of tissue samples obtained from lung cancer patients to further confirm the expression level of UCHL3 in lung cancer. The UCHL3 protein was detected in both lung cancer tissues and paracancerous normal tissues, and its expression was significantly elevated in lung ADC and SCC (Fig. 1c). Moreover, we found that immunohistochemical scores (IHSs) for UCHL3 in lung ADC and SCC tissues were higher than those in paracancerous normal tissues (Fig. 1d). We used Kaplan–Meier analysis to evaluate the relationship between the expression level of UCHL3 and the survival rate of NSCLC patients. The results indicated that high expression of UCHL3 corresponded with a low survival rate (Fig. 1e). In addition, we performed Kaplan–Meier analysis in other cancer types, breast cancer (BRCA), kidney cancer (KIRC) and liver cancer (LIHC), which showed that UCHL3 levels were upregulated in these cancers (Supplementary Fig. S1a). In addition, high UCHL3 levels were associated with poor overall survival (Supplementary Fig. S1b). We observed that both AhR and UCHL3 protein levels were increased in lung cancer tissues compared with normal tissues, and a positive correlation between AhR and UCHL3 protein levels was observed (Supplementary Fig. S1c). Our results show that UCHL3 in the cytoplasm may play a biological role and that its expression may be increased in human cancers.

### Overexpression of UCHL3 promotes cell growth, colony formation, tumor formation, and tumor stem-like properties

**Fig. 2:**
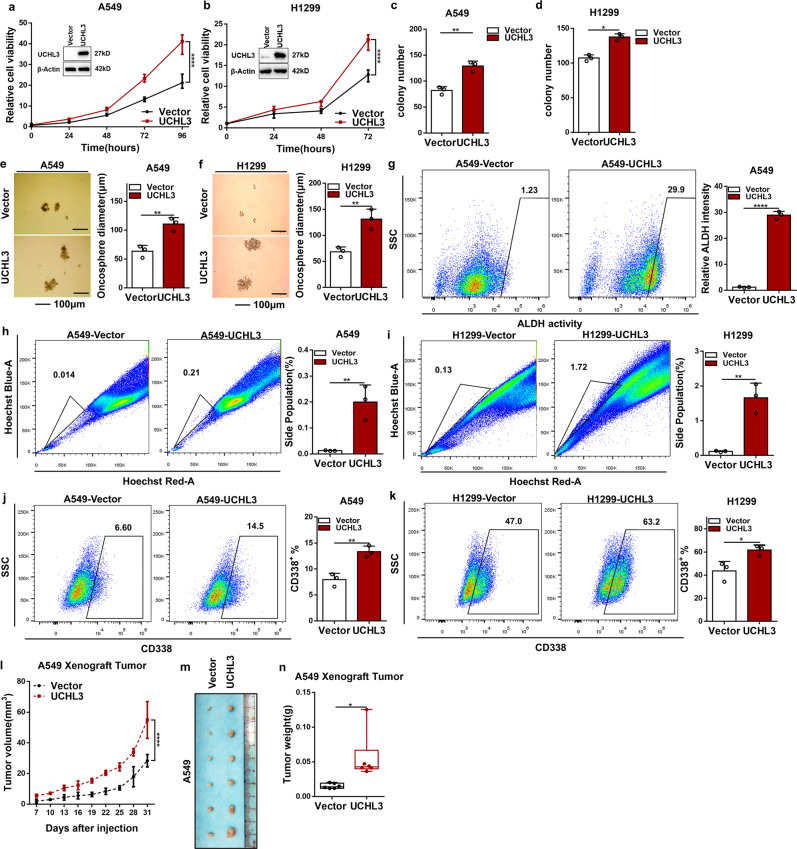
Overexpression of UCHL3 promoted cell growth, colony formation, tumor formation, and tumor stem-like properties. **a**, **b** The MTS assay was used to assess cell viability in A549 (**a**) and H1299 (**b**) cells stably overexpressing UCHL3 (n=5). Data are shown as the mean ± SD; ****p < 0.0001. **c**, **d** A colony formation assay in plates was performed to detect the colony formation ability of A549 (**c**) and H1299 (**d**) cells stably overexpressing UCHL3 (*n* = 3); representative images are shown in the Supplementary section, and the results show that UCHL3 promotes colony formation. Data are shown as the mean ± SD; *p < 0.05, **p < 0.01. **e**, **f** UCHL3 was overexpressed in A549 (**e**) and H1299 (**f**) cells seeded in ultralow attachment dishes to allow tumor sphere formation, and the results are shown as a bar graph (*n* = 3, scale bar = 100 μm). Data are shown as the mean ± SD; **p < 0.01. **g** Flow cytometry analysis of ALDH activity in A549 cells overexpressing UCHL3 (*n* = 3). Data are shown as the mean ± SD; ****p < 0.0001. **h**, **i** Flow cytometry analysis showing side populations of cells among the A549 (**h**) and H1299 (**i**) cell lines overexpressing UCHL3, with the results shown as a bar graph (*n* = 3). Data are shown as the mean ± SD; **p < 0.01. **j**, **k** Representative images from flow cytometry analysis to detect CD338-positive A549 (**j**) and H1299 (**k**) cells overexpressing UCHL3, with the results shown as a bar graph (*n* = 3). Data are shown as the mean ± SD; *p < 0.05, **p < 0.01. **l–n** A xenograft model of tumor growth was established to evaluate the ability of A549 cells with stable UCHL3 overexpression to form tumors (*n* = 6 mice per group). Tumor formation was monitored at the indicated times (**l**), and images (**m**) and weight (**n**) are presented (*n* = 6). Data are shown as the mean ± SD; *p < 0.05, ****p < 0.0001. All data in bar graphs were assessed by two-tailed Student’s *t*-test

According to Kaplan–Meier analysis, compared with its impact on the clinical prognosis of SCC patients (*P* = 0.6641), the expression of UCHL3 had a greater impact on the clinical prognosis of ADC patients (*P* = 0.0157). Therefore, ADC cell lines were selected as research models. Then, we determined the UCHL3 expression levels in a group of lung ADC cell lines (Supplementary Fig. S1d). UCHL3 exhibited low expression levels in A549 and H1299 cell lines and high expression levels in the H358 cell line. Next, using lentivirus, UCHL3 was overexpressed in A549 and H1299 cells and knocked down in H358 cells. We used western blotting to test the overexpression and knockdown efficiency. UCHL3 overexpression enhanced A549 and H1299 cell growth in vitro (Fig. 2a, b). Moreover, UCHL3 overexpression in the A549 and H1299 cell lines obviously increased colony formation ability (Fig. 2c, d and Supplementary Fig. S2a, b). Furthermore, both UCHL3-overexpressing cell lines showed more stem-like characteristics, such as spheroid growth ability (Fig. 2e, f) and ALDH activity, as shown by ALDEFLUOR assays (Fig. 2g), than vector cells. As functional markers of cancer stem cells, side populations (SPs) in the cell lines show stem-like properties. We used flow cytometry to detect the percentage of SP cells in vector-transfected and UCHL3-overexpressing cells and found that UCHL3-overexpressing cells had a higher percentage of SP cells (Fig. 2h, i). Moreover, flow cytometry analysis showed that the percentage of CD338-positive cells among UCHL3-overexpressing cells was higher than that among vector-transfected cells (Fig. 2j, k).

To further investigate the effect of UCHL3 on tumor formation in vivo, we performed a xenograft model experiment. Compared with the injection of vector-transfected A549 cells, injection of UCHL3-overexpressing A549 cells significantly increased tumor size, volume, and weight after 1 month of growth (Fig. 2l–n), while the overall body weight was maintained (Supplementary Fig. S2c). These results conclusively show that UCHL3 overexpression is related to cell growth, colony formation, spheroid formation, and tumor growth and that UCHL3 elevates stem-like properties and has critical oncogenic functions in cancer progression.

### Knockdown of UCHL3 inhibited cell growth, colony formation, tumor formation, and tumor stem-like properties

**Fig. 3:**
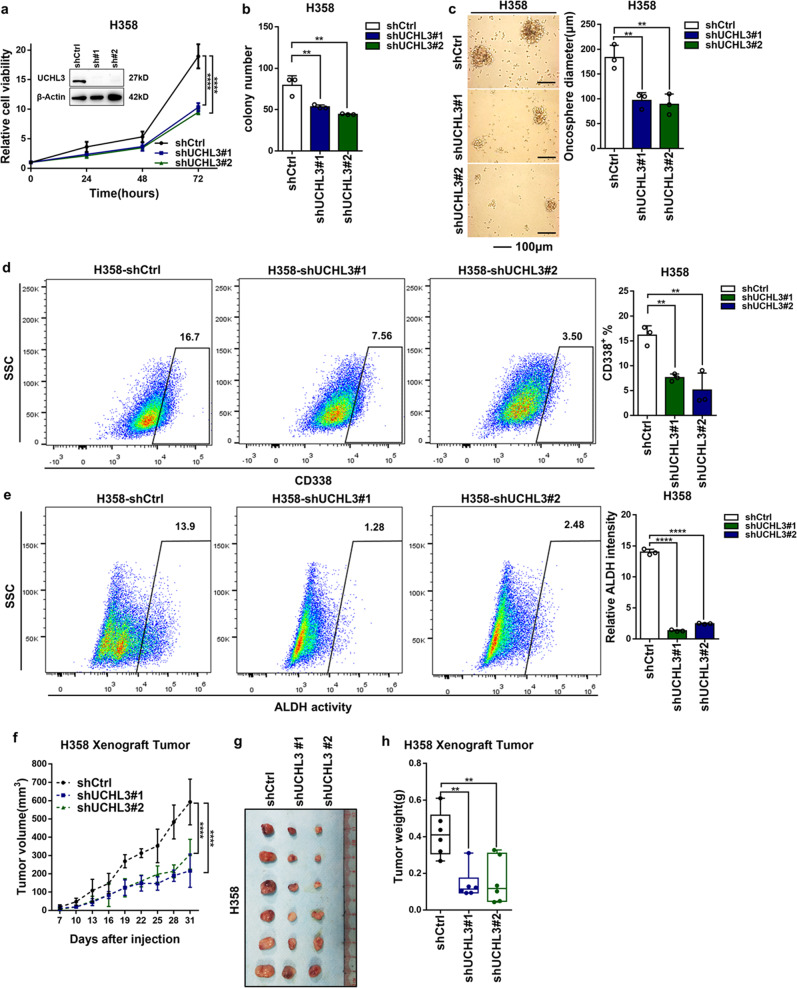
Knockdown of UCHL3 inhibited cell growth, colony formation, tumor formation, and tumor stem-like properties. **a The** MTS assay was used to assess cell viability in H358 cells with stable UCHL3 knockdown (*n* = 5). Data are shown as the mean ± SD; ****p < 0.0001. **b** A colony formation assay in plates was performed to detect the colony formation ability of H358 cells with stable UCHL3 knockdown (*n* = 3); representative images are shown in the Supplementary section, and the results show that knockdown of UCHL3 inhibited colony formation. Data are shown as the mean ± SD; **p < 0.01. **c** The H358 cell line with UCHL3 knockdown was seeded in ultralow attachment dishes to allow tumor sphere formation, and the results are shown as a bar graph (*n* = 3, scale bar = 100 μm). Data are shown as the mean ± SD; **p < 0.01. **d** Representative images from flow cytometry analysis to detect CD338-positive cells among UCHL3-knockdown H358 cells, with the results shown as a bar graph (*n* = 3). Data are shown as the mean ± SD; **p < 0.01. **e** Flow cytometry analysis of ALDH activity in the UCHL3-knockdown H358 cell line, with the results shown as a bar graph (*n* = 3). Data are shown as the mean ± SD; ****p < 0.0001. **f–h** A xenograft model of tumor growth was established to evaluate the ability of H358 cells with stable UCHL3 knockdown to form tumors. Tumor formation was monitored at the indicated times (**f**), and images (**h**) and tumor weights (**g**) were recorded (*n* = 6 mice per group). Data are shown as the mean ± SD; **p < 0.01, ****p < 0.0001. Data in all bar graphs were assessed by one-way ANOVA with multiple comparisons

To reveal the physiological role of UCHL3 in lung cancer, we stably knocked down UCHL3 in H358 cells. Four different UCHL3-targeting shRNAs were applied to knock down UCHL3 expression, and shUCHL3#1 and shUCHL3#2 achieved a knockdown efficiency of ~100%. Therefore, unless otherwise specified, shUCHL3#1 and shUCHL3#2 were used in subsequent studies. As expected, the growth rate of UCHL3-knockdown cells was significantly slower than that in control cells (Fig. 3a). Additionally, the knockdown of UCHL3 markedly restrained the colony formation ability of the cells (Fig. 3b and Supplementary Fig. S3a), and H358 cells depleted of UCHL3 showed fewer stem-like characteristics in the spheroid formation experiment (Fig. 3c). Flow cytometry analysis indicated that knockdown of UCHL3 reduced the proportion of CD338^+^ H358 cells (Fig. 3d). By ALDEFLUOR analysis, after UCHL3 depletion, the ALDH activity in H358 cells was decreased (Fig. 3e), suggesting that UCHL3 promotes stem-like properties in lung cancer.

To investigate in vivo tumor formation, H358 cells were injected into nude mice, and we found that UCHL3 depletion significantly reduced tumor size, volume, and weight (Fig. 3f–h); however, there was no significant difference in body weight between the two groups (Supplementary Fig. S3b). In general, these results indicated that UCHL3 promotes cancer stem-like properties via an oncogenic function.

### UCHL3 interacts with AhR and stabilizes the AhR protein through deubiquitination

**Fig. 4:**
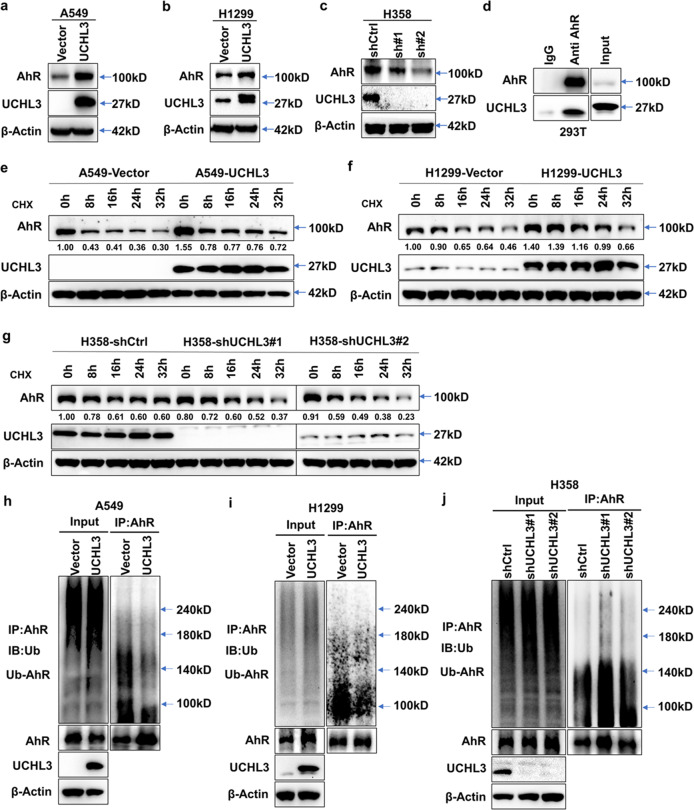
UCHL3 interacts with AhR and stabilizes the AhR protein through deubiquitination. **a–c** Western blot analysis was used to detect the expression level of AhR in A549 (**a**), H1299 (**b**) and H358 (**c**) cells after overexpression or depletion of UCHL3. **d** Exogenous UCHL3 and AhR proteins interacted in HEK293T cells. AhR and UCHL3 were coexpressed in HEK293T cells, and the AhR protein was immunoprecipitated with anti-AhR antibody. IgG served as a negative control, and exogenous UCHL3 was detected by WB. **e**, **f** UCHL3 overexpression delayed AhR protein degradation. After the treatment of UCHL3-overexpressing A549 (**e**) and H1299 (**f**) cells with cycloheximide (CHX, 10 μg/ml) for the indicated durations, AhR protein expression was analyzed by WB. Quantification of the AhR protein band was performed using ImageJ software. **g** UCHL3 knockdown enhanced AhR protein degradation. After UCHL3 knockdown, H358 cells were treated with cycloheximide (CHX, 10 μg/ml) for the indicated duration, and AhR protein expression was analyzed by WB. Quantification of the AhR protein band was performed using ImageJ software. **h, i** The lysates of A549 (**h**) and H1299 (**i**) cells stably overexpressing UCHL3 and vector-transfected cells containing 1 mg of total protein for each panel were immunoprecipitated with 2 μg of anti-AhR antibody, following which AhR ubiquitination was examined using anti-Ub antibody. **j** The lysates of stable UCHL3-knockdown H358 cells and shCtrl-transfected cells containing 1 mg of total protein were immunoprecipitated with 2 μg of anti-AhR antibody, and AhR ubiquitination was examined using anti-Ub antibody

Our previous studies indicated that irradiation-resistant (IR) cells show increased stem-like characteristics with enhanced growth and metastasis capacity.^26,39^ To investigate whether UCHL3 regulates biological functions through AhR, we used Western blotting to determine whether expression levels of the AhR protein in A549 and H1299 cells were upregulated after UCHL3 overexpression (Fig. 4a, b). Furthermore, in stable UCHL3-knockdown H358 cells, expression of the AhR protein was downregulated (Fig. 4c), indicating that UCHL3 plays a key role in regulating AhR expression in lung cancer. To determine whether UCHL3 stabilizes the AhR protein through deubiquitination, we first examined AhR protein degradation through the ubiquitin-proteasome system by using cycloheximide (CHX) and traced the protein levels. Indeed, the AhR protein was gradually degraded with CHX treatment (Supplementary Fig. S4a). Then, we explored the interaction between the UCHL3 and AhR proteins. AhR and UCHL3 plasmids were cotransfected into HEK293T cells for protein–protein interaction analysis via coimmunoprecipitation, and we discovered that the UCHL3 protein coimmunoprecipitated with AhR (Fig. 4d), indicating a relationship between UCHL3 and AhR. To further determine whether UCHL3 can protect the AhR protein by delaying its degradation, we overexpressed UCHL3 in A549 and H1299 cells, knocked down UCHL3 in H358 cells using two different shRNAs and detected the AhR protein degradation rate by cycloheximide (CHX) chase assay. As anticipated, when UCHL3 was overexpressed, AhR protein degradation was reduced (Fig. 4e, f). The AhR protein half-life was decreased after depletion of UCHL3 (Fig. 4g). To directly detect whether UCHL3 regulates AhR protein stabilization via deubiquitination, endogenous polyubiquitinated AhR protein was immunoprecipitated with anti-AhR antibody and examined using anti-Ub antibody. Notably, in cells not treated with the proteasome inhibitor MG132, AhR was not protected from degradation.^40,41^ Furthermore, overexpression of UCHL3 decreased the ubiquitination of endogenous AhR in A549 and H1299 cells (Fig. 4h, i), while depletion of endogenous UCHL3 elevated the ubiquitination level of endogenous AhR in H358 cells (Fig. 4j). In summary, our experiments indicate that UCHL3 is a specific DUB for AhR and that UCHL3 elevates AhR protein stabilization through deubiquitination.

We further detected mRNA levels in 35 paired ADC and SCC lung cancer and corresponding paracancerous normal tissues (Supplementary Fig. S4b, c) and noted no difference between histologic subtypes. AhR protein levels were markedly upregulated in ADC and SCC lung cancer tissues compared with corresponding paracancerous normal tissues (Supplementary Fig. S4d, e). Moreover, Kaplan–Meier curves showed that patients expressed different AhR mRNA levels but showed no difference in prognosis (Supplementary Fig. S4f), indicating that high AhR expression in lung cancer is mainly due to posttranslational regulation.

### UCHL3 promotes tumor stem-like properties through AhR

**Fig. 5:**
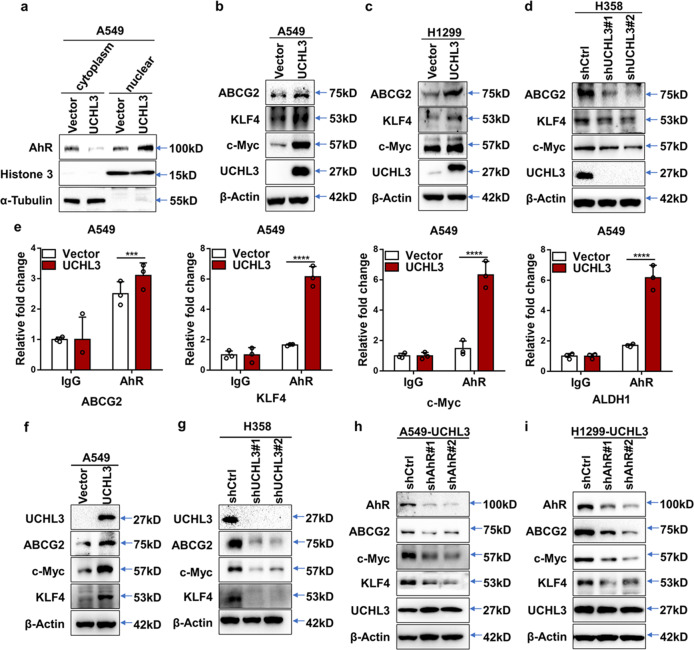
UCHL3 promotes tumor stem-like properties through AhR. **a** Western blot analysis was used to detect AhR levels in nuclear and cytosolic fractions derived from A549 cells overexpressing UCHL3. **b–d** Western blot analysis was used to detect stemness-associated markers in A549 (**b**) and H1299 (**c**) cells overexpressing UCHL3 or UCHL3-knockdown H358 cells (**d**). **e** ChIP analysis was performed in A549 cells overexpressing UCHL3 to detect AhR binding to stemness-related genes as indicated (*n* = 3). Data are shown as the mean ± SD; two-way ANOVA with multiple comparisons; ***p < 0.001, ****p < 0.0001. **f**, **g** Western blot analysis was used to detect stemness-associated markers in (**f**) xenograft tumors from A549 cells overexpressing UCHL3 and (**g**) H358 cells with UCHL3 knockdown. **h**, **i** Western blot analysis was used to detect stemness-associated markers in UCHL3-overexpressing A549 (**h**) and H1299 (**i**) cells transiently transfected with AhR-knockdown shRNA plasmid or blank plasmid. Cell lysates were harvested at 48 h

In our previous studies, we showed that AhR promotes lung cancer cell irradiation resistance and tumor stem-like properties.^26^ As UCHL3 is a DUB of AhR, we sought to understand more about UCHL3-mediated regulation of tumor stem-like properties. We searched for similar molecular mechanisms in cell lines expressing UCHL3. Since AhR is located in both the cytoplasm and nucleus, we separately assessed the expression of UCHL3 in the cytoplasm and nucleus after fractionation and discovered that the nuclear localization of AhR was elevated in cells overexpressing UCHL3 compared to control cells (Fig. 5a; Supplementary Fig. S4g), while knockdown of UCHL3 had the opposite result (Supplementary Fig. S4h), indicating that AhR might play a regulatory role in the expression of stemness genes. As expected, overexpression of UCHL3 increased the expression of ABCG2, c-Myc and KLF4, genes associated with stemness, at the protein level in A549 and H1299 cells (Fig. 5b, c), while depletion of UCHL3 decreased their protein expression (Fig. 5d). Moreover, we found that AhR could bind the promoter regions of stemness-related genes using ALGGEN (http://alggen.lsi.upc.es/). ChIP assays showed that AhR was recruited to the promoters of the stemness-related genes ABCG2, KLF4, c-Myc and ALDH1 in A549 cells and that the enrichment of their promoter regions in AhR was greater in A549 cells overexpressing UCHL3 than in control cells (Fig. 5e). Finally, we determined that stemness-associated markers were significantly upregulated in xenograft tumor samples with UCHL3 protein knockdown compared with the control groups (Fig. 5f, g). To determine whether UCHL3-medaited control of the levels of stemness-related genes is dependent on AhR, we tested these genes in the absence of AhR by transient transfection with AhR-knockdown shRNA plasmids and blank plasmid. Our results demonstrated that in the absence of AhR, expression of the stemness-related genes ABCG2, c-Myc and KLF4 was decreased. Thus, the overexpression of UCHL3 controls the levels of these genes in a manner dependent on AhR (Fig. 5h, i). In summary, our findings revealed that AhR plays a critical role in maintaining the stem-like properties of cells overexpressing UCHL3, confirming that UCHL3 promotes tumor stem-like properties through stabilizing AhR.

### UCHL3 increases AhR protein stability in a DUB activity-dependent manner

**Fig. 6:**
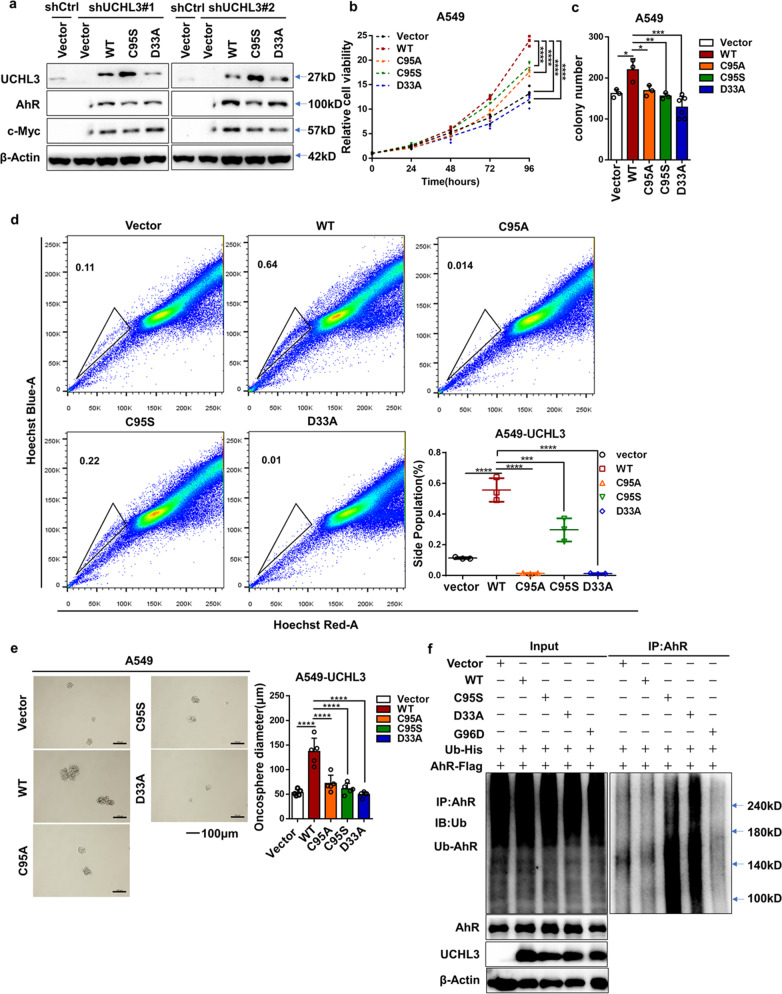
UCHL3 increases AhR protein stability in a DUB activity-dependent manner. **a** UCHL3 stable knockdown by shRNA#1 and shRNA#2 in H358 cells induced changes in AhR and c-Myc protein expression that were rescued by transient overexpression of UCHL3 but not UCHL3-C95S and UCHL3-D33A. **b** The MTS assay was used to assess cell viability in A549 cells stably overexpressing UCHL3 and UCHL3 point mutants(*n* = 5). Data are shown as the mean ± SD; ****p < 0.0001. **c** Colony formation assays were performed to detect the colony formation ability of A549 cells stably overexpressing UCHL3 and UCHL3 point mutants (*n* = 3); representative images are shown in the Supplementary section. Data are shown as the mean ± SD; *p < 0.05, **p < 0.01, ***p < 0.001. **d** Flow cytometry analysis showing side populations among A549 cells stably overexpressing UCHL3 and UCHL3 point mutants, with the results shown as a bar graph (*n* = 3). Data are shown as the mean ± SD; ***p < 0.001, ****p < 0.0001. **e** A549 cells stably overexpressing UCHL3 and UCHL3 point mutants were seeded in ultralow attachment dishes to allow tumor sphere formation, and the results are shown as a bar graph (n=5, scale bar = 100 μm). Data are shown as the mean ± SD; ****p < 0.0001. **f** UCHL3 decreased AhR ubiquitination in HEK293T cells. AhR-Flag and Ub-His were coexpressed with vector, UCHL3, UCHL3-C95S, UCHL3-D33A or UCHL3-G96D in HEK293T cells. Cell lysates were harvested after 72 h, AhR proteins were immunoprecipitated with anti-AhR antibody, and polyubiquitinated AhR proteins were detected by WB using anti-Ub antibody. Data in all bar graphs were assessed by one-way ANOVA with multiple comparisons

Analysis of UCHL3 mutants showed that the aspartic acid residue at position 33 and the cysteine residue at position 95 are key sites for the interaction between UCHL3 and ubiquitin.^42^ To restore the knockdown effects of UCHL3 shRNAs, we re-expressed wild-type UCHL3 and the inactive mutants UCHL3-C95S and UCHL3-D33A in H358 cells with stable UCHL3 knockdown. WT UCHL3 obviously increased AhR and c-Myc protein levels, whereas the mutants did not (Fig. 6a). We also found that some UCHL3 point mutants affected the UCHL3 protein level (Supplementary Fig. S5a). Interestingly, stable overexpression of UCHL3 mutants in A549 cells significantly attenuated cell growth compared to that in cells expressing WT UCHL3 (Fig. 6b). FACS analysis showed that the UCHL3 mutants reduced the number of SP cells among A549 cells (Fig. 6c). Moreover, UCHL3 knockdown significantly suppressed colony formation ability (Fig. 6d and Supplementary Fig. S5b). A549 cells expressing UCHL3 mutants showed fewer stem-like characteristics, as shown by their decreased ability to form spheroids (Fig. 6e), suggesting that UCHL3 stabilizes the AhR protein in a DUB activity-dependent manner. In addition, we cotransfected HEK293T cells with AhR, Ub and WT UCHL3 or the UCHL3-C95S, UCHL3-D33A and UCHL3-G96D mutants. The levels of polyubiquitinated AhR protein were tested by using anti-Ub antibody. WT UCHL3, but not UCHL3-C95S, significantly reduced AhR protein polyubiquitination (Fig. 6f), whereas the other mutants did not decrease AhR polyubiquitination. Generally, these findings indicated that as a DUB of AhR, UCHL3 increases AhR protein stabilization in a DUB activity-dependent manner.

### Inhibition of UCHL3 weakened cancer stem cell properties

**Fig. 7:**
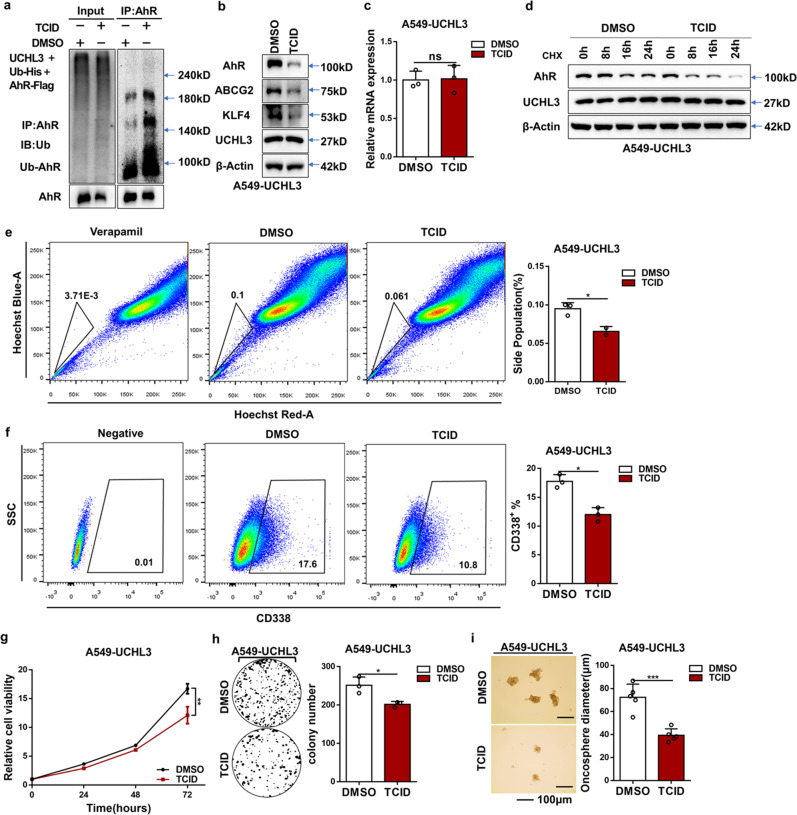
Inhibition of UCHL3 weakened cancer stem cell properties, as noted in a schematic model of the effects of UCHL3 on tumorigenesis and stem-like properties. **a** TCID decreased AhR ubiquitination in HEK293T cells by inhibiting UCHL3 activity. AhR-Flag and Ub-His were coexpressed with vectors in HEK293T cells, and cells were treated with DMSO and TCID (10 μM) for 24 h. Cell lysates were harvested after 72 h. The AhR protein was immunoprecipitated, and polyubiquitinated AhR protein was detected by WB using anti-Ub antibody. **b** Western blot analysis was used to detect stemness-associated markers in UCHL3-overexpressing A549 cells treated with DMSO or TCID (10 μM). **c** RT-qPCR was used to detect AhR mRNA levels in UCHL3-overexpressing A549 cells treated with DMSO or TCID (10 μM) (*n* = 3). Data are shown as the mean ± SD; ns indicates nonsignificant (*p* > 0.05). **d** TCID accelerated AhR protein degradation. After cotreatment of A549 cells overexpressing UCHL3 with DMSO/TCID (10 μM) for 24 h and cycloheximide (CHX, 10 μg/ml) for the indicated duration, AhR protein expression was analyzed by WB. **e** Flow cytometry analysis showing side populations among A549 cells stably overexpressing UCHL3 treated with DMSO or TCID (10 μM) for 24 h, with the results shown as a bar graph (*n* = 3). Data are shown as the mean ± SD; *p < 0.05. **f** Representative images of flow cytometry analysis to detect CD338-positive cells among A549 cells stably overexpressing UCHL3 treated with DMSO or TCID (10 μM) for 24 hours, with the results shown as a bar graph (*n* = 3). Data are shown as the mean ± SD; *p < 0.05. **g** The MTS assay was used to assess the cell viability of A549 cells stably overexpressing UCHL3 treated with DMSO or TCID (10 μM) (*n* = 5). Data are shown as the mean ± SD; **p < 0.01. **h** Colony formation assays were performed to detect the colony formation ability of A549 cells stably overexpressing UCHL3 treated with DMSO or TCID (10 μM) (*n* = 3); representative images are shown on the side. Data are shown as the mean ± SD; *p < 0.05. **i** A549 cells stably overexpressing UCHL3 treated with DMSO or TCID (10 μM) were seeded in ultralow attachment dishes to allow tumor sphere formation, with the results shown as a bar graph (*n* = 5, scale bar = 100 μm). Data are shown as the mean ± SD; ***p < 0.001. Data in all bar graphs were assessed by two-tailed Student’s *t*-test

**Fig. 8:**
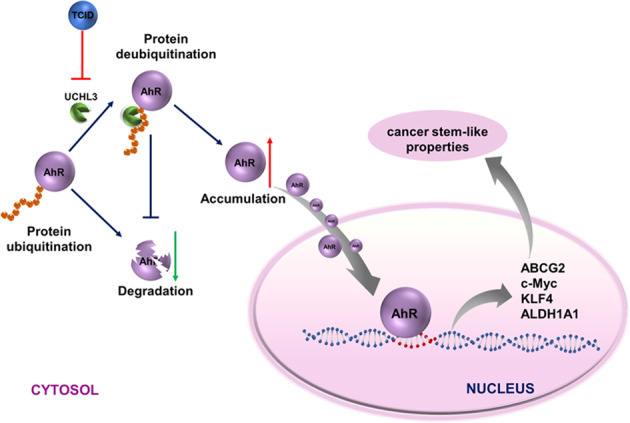
A model of AhR regulation by UCHL3. UCHL3, a DUB, promotes stem-like properties by increasing AhR protein levels in an activity-dependent manner. Consequently, AhR recruitment to the promoters of stemness-related genes induces the expression of genes in a stem-like gene signature

To further demonstrate our findings, we used TCID, a small-molecule inhibitor of UCHL3; under our experimental conditions, we first detected whether TCID inhibits the deubiquitinase activity of UCHL3 against AhR. As expected, the ability of UCHL3 to deubiquitinate AhR was almost completely abrogated by TCID treatment (Fig. 7a). Correspondingly, TCID treatment reduced AhR protein levels and stemness-associated markers in UCHL3-overexpressing A549 cells without affecting AhR mRNA levels (Fig. 7b, c), suggesting that TCID, like UCHL3 knockdown, promotes AhR ubiquitination and degradation. Moreover, cotreatment with TCID and CHX significantly shortened the half-life of the AhR protein (Fig. 7d). FACS analysis showed that inhibition of UCHL3 using TCID reduced CD338 expression and the percentage of SP cells among A549 cells overexpressing UCHL3 (Fig. 7e, f). We also found that treatment of UCHL3-overexpressing cells with TCID markedly suppressed cell proliferation and colony formation ability (Fig. 7g, h). Inhibition of UCHL3 reduced stem-like characteristics, as shown by their decreased ability to form spheroids (Fig. 7i), suggesting that UCHL3 promotes stem-like characteristics in lung cancer. Collectively, these findings indicate that inhibition of UCHL3 may effectively eliminate lung cancer stem cell properties by promoting AhR destabilization. In view of our findings, we designed a model (Fig. 8) in which UCHL3 acts as a novel DUB of AhR that participates in tumorigenesis and stem-like properties.

## Discussion

The transcription factor AhR promotes cancer cell cycle progression, survival, migration, stemness and tumorigenesis.^43–46^ AhR is a primary factor affecting tumorigenesis and plays an important role in breast CSCs.^47,48^ A better comprehension of the AhR upstream regulatory mechanism may reveal new therapeutic targets for AhR-positive cancers. DUBs can reverse ubiquitination. We performed a series of experiments and identified UCHL3 as a DUB of AhR. First, UCHL3 and AhR directly interact. Second, UCHL3 reduces AhR polyubiquitination and enhances AhR protein stabilization in a DUB activity-dependent manner, and this ability could be inhibited by TCID, a UCHL3 inhibitor. Finally, UCHL3 functions to promote tumor growth and lung cancer stem-like properties through AhR.

Disorder in the deubiquitination process is usually related to tumorigenesis.^6^ Deubiquitination regulates the functions of many tumorigenesis-associated proteins, such as p53, Mdm2, Sirt1 and PTEN.^49–53^ Cancer stem cells (CSCs) are cancer cells with an infinite capacity to proliferate, giving them stem cell-like characteristics, and CSCs play a key role in tumor recurrence, resistance and progression.^54^ The transcription factor network, CSC-associated proteins and the microenvironment are three major factors that affect CSC maintenance and differentiation.^55,56^ The ubiquitination and deubiquitination of proteins associated with stemness are very important for the maintenance and differentiation of CSCs. Furthermore, the ubiquitination and deubiquitination of some crucial proteins in stem cells can determine cell fate.^57,58^ Recently, DUBs have been shown to be promising cancer therapeutic targets,^59–61^ but their roles in cancer cell stemness remain unclear. The identification of deubiquitylases of transcription factors and key proteins can improve comprehension of the activation mechanism in CSCs.

Many DUBs are involved in cancer progression in a variety of organs. Nevertheless, until recently, the mechanism and role of UCHL3 in carcinogenesis have remained elusive. UCHL3 may play a critical role in promoting epithelial-to-mesenchymal transition (EMT) and DNA damage repair.^42,62–64^ A limited number of studies on the role of UCHL3 in tumors have been conducted, and very few UCHL3 targets have been identified. In this study, we showed that UCHL3 overexpression increased tumor stem-like properties. Stemness-related genes and other stemness markers were elevated in lung cancer cells overexpressing UCHL3. Importantly, stable knockdown of UCHL3 suppressed tumor growth from H358 cells and inhibited tumor stem-like properties. Moreover, TCID attenuated UCHL3 activity without affecting its protein level. Thus, TCID can significantly reduce AhR protein levels and diminish tumor stem-like properties. Nevertheless, our results suggest that WT UCHL3, but not UCHL3 mutants, promotes tumor growth and stem-like properties through stabilizing AhR in a DUB activity-dependent manner. The mechanism by which UCHL3 promotes tumor stem-like properties through AhR requires further investigation.

In conclusion, UCHL3 is an AhR DUB that promotes lung cancer proliferation, tumor growth and tumor stem-like properties through stabilizing AhR by its deubiquitination. In this paper, we found a new mechanism of AhR regulation and functional mechanism by which UCHL3 promotes lung cancer progression and stemness. Our research indicates that UCHL3 might serve as a therapeutic target in lung cancer.

## Materials and methods

### Cell culture, antibodies, plasmids, siRNAs, and chemicals

The following NSCLC cell lines were used in this paper: A549 (ATCC: CCL-185™), H1299 (ATCC: CRL-5803™) and H358 (ATCC: CRL-5807™). All cell lines were purchased from ATCC. The following media were required for cell culture: 1:1 DME/F12 (HyClone, UT, USA) for the A549 cell line, DMEM (Gibco, NY, USA) for the HEK293T cell line, and RPMI1640 (Gibco) for other cell lines. Cells were cultured in a cell incubator at 37 °C with 5% CO_2_, and 10% (v/v) FBS was added to the medium. Mycoplasma contamination was detected and shown to be negative in all cell lines, and only cells that had been passaged no more than 10 times after initial resuscitation from cryopreservation were used. All cell lines were identified by short tandem repeat analysis prior to use. UCHL3 cDNA clones were purchased from Vigene Biosciences. The FLAG-UCHL3 overexpression plasmid was established by insertion of the UCHL3 cDNA into the pLVX-EF1α-IRES-Puro vector (catalog No. 631988; Clontech, CA, USA). The primers used in this paper are listed in Supplementary Table 2. Targeted and nontargeted control vector lentiviral shRNA clones were established by GeneChem (www.genechem.com.cn) (Shanghai, China). The sequences of shRNAs targeting UCHL3 and AhR used in this paper are presented in Supplementary Table 1. Plasmid transfection using Lipofectamine® 2000 was carried out according to the manufacturer’s instructions, and colonies with stable expression were screened by puromycin (2 μg/ml). Cycloheximide (CHX, Sigma-Aldrich, MO, USA) And TCID (B1467, APExBIO, TX, USA) were also used in the cell experiments.

### Cell proliferation and colony formation assays

In the cell proliferation assay, the CellTiter 96 AQueous One Solution Cell Proliferation Assay (MTS, 3-(4,5-dimethylthiazol-2-yl)-5-(3-carboxymethoxyphenyl)-2-(4-sulfophenyl)-2H-tetrazolium) kit (Promega, WI, USA) was used in accordance with its instructions. First, each well of a 96-well plate was seeded with 500 cells. Measurement of the OD_450_ began 1 h after mixing with MTS reagent. In the cell colony formation experiment, each of the wells of six-well plates was seeded with approximately 500 cells that were then cultured in a cell incubator. Two weeks later, the cells were fixed with methanol and stained with 0.5% crystal violet. Clones were counted by using a microscope and ImageJ software (1.47 v, NIH, USA).

### Oncosphere formation assay

Cells were cultured in ultralow attachment culture dishes (Corning, NY, USA) with serum-free DMEM-F12 medium containing insulin (50 μg/ml final concentration, Sigma-Aldrich, MO, USA), albumin (bovine) fraction V (0.4% final volume concentration, Sigma-Aldrich, MO), N-2 Plus Media Supplement (Life Technologies, NY, USA), B-27 Supplement (Life Technologies, NY, USA), EGF (20 μg/ml final concentration, PeproTech, NJ, USA), and basic FGF (10 μg/ml final concentration, PeproTech, NJ, USA) to sustain the growth of undifferentiated oncospheres. After the cells had been continuously incubated in a cell incubator for 1–2 weeks, cells in oncospheres were counted under a microscope.

### Chromatin immunoprecipitation (ChIP) assays

In general, the ChIP assay was performed as described before^65,66^ with minor modifications. Formaldehyde (1% final volume concentration, Sigma-Aldrich, MO, USA) was used to fix the cells (5 × 10^6^) at temperature for 10 min. Then, 1.25 M glycine was added and incubated at room temperature for 5 min to terminate fixation. Ultrasonication was carried out in a Qsonica sonicator for 6 min (20 s on; 20 s off), with each immunoprecipitation assay using 300 μg of protein-chromatin complex. The antibody-protein complex was captured using preblocked Dynabeads protein G (10004D, Thermo Fisher Scientific, MA, USA). ChIP DNA was detected in an ABI 7500 real-time PCR instrument (Applied Biosystems, CA, USA) using SYBR Green (Bio-Rad, CA, USA). The primers used in this assay are listed in Supplemental Table 2. The antibody used was anti-AhR (ab126703, Abcam, MA, USA) antibody.

### Western blot analysis and coimmunoprecipitation (Co-IP) assay

Collected cells were washed three times in 1x PBS and then lysed on ice in IP lysis buffer containing protease inhibitor cocktail. An SDS-polyacrylamide gel was used to separate the total proteins obtained by cell lysis, which were then transferred to a polyvinylidene fluoride membrane. The primary antibodies used for western blot analysis are listed below. Protein detection was performed using mouse monoclonal anti-β-actin (A5441, Sigma-Aldrich, MO, USA), rabbit monoclonal anti-UCHL3 (8141P, Cell Signaling Technology, MA, USA), rabbit monoclonal anti-c-Myc (5605, Cell Signaling Technology, MA, USA), mouse polyclonal anti-AhR (sc-133088, Santa Cruz, CA, USA), mouse monoclonal anti-ABCG2 (MAB4146, Sigma-Aldrich, MO, USA), rabbit polyclonal anti-KLF4 (sc-20691×, Santa Cruz, CA, USA), rabbit polyclonal anti-histone-H3 (17168-1-AP, Proteintech, IL, USA), and mouse monoclonal anti-α-tubulin (sc-5286, Santa Cruz, CA, USA).

For immunoprecipitation experiments, cells were cultured overnight in culture dishes (~1.5 × 10^6^ cells per dish). Then, the cells were harvested and lysed in IP buffer, and 20 μL of Dynabeads protein G (10004D, Thermo Fisher Scientific, MA, USA) was added to 1 mg of total protein, stirred and incubated at 4 °C for 2 h. The supernatant was then collected after centrifugation at 2000 rpm for 10 min. The collected supernatant was subjected to mild rotation in the presence of 1x protease inhibitors and incubated with 2 μg of anti-AhR antibody (sc-133088, Santa Cruz, CA, USA) at 4 °C overnight. The precipitated protein complex was recovered with a magnetic rack, and then the beads were washed with cold immunoprecipitation assay buffer three times. The beads collected from the procedure above were mixed with IP buffer and protein loading buffer. Then, the cells were boiled at 100 °C for 5 min to free the bound protein. Total protein (20 μg) was used as an input control. Samples were analyzed by western blotting with antibodies against AhR and UCHL3.

### ALDEFLUOR assay and flow cytometry

In accordance with the manufacturer’s protocol, cells were stained with antibodies or fluorescent dyes (CD338-PerCP-Cy™5.5, BD Bioscience, CA, USA; Hoechst 33342, Sigma-Aldrich, MO, USA) before being loaded for flow cytometry. A FACSCalibur (BD Immunocytometry Systems, CA, USA) was applied to detect the labeled cells, and the resulting analysis was performed in FlowJo software. IgG isotype controls were used to eliminate background due to the nonspecific binding of antibodies to the cell surface to accurately set the threshold for negative and positive cells. Cell clusters and debris were removed by lateral scattering and forward scattering analysis. With the guidance and help of Dr. Suling Liu (Fudan University, Shanghai, China), an ALDEFLUOR kit (STEMCELL Technologies, Vancouver, BC, Canada) was utilized as described before.^67^

### RT-qPCR

TRIzol reagent (Takara, Kusatsu, Japan) was used to separate total RNA, and a PrimeScriptTM RT reagent kit (Takara, Kusatsu, Japan) containing gDNA Eraser (Perfect Real Time) was used to generate cDNA. Real-time PCR was run in an ABI 7500 Real-time PCR instrument using FastStart Universal SYBR Green Master. Relative gene expression was standardized by β-actin.

### Histology and immunohistochemistry

The Department of Pathology at Xiangya Hospital verified and provided biopsies of lung cancer and associated diseases. The method used for IHC analysis of paraffin sections from lung cancer tissues can be found in previous literature. Images of the paraffin sections were captured with a CX41 microscope (Olympus, Tokyo, Japan) equipped with a DP-72 microscope digital camera system (Olympus, Tokyo, Japan), and differential quantification was performed by two pathologists from Xiangya Hospital, Changsha, China.

### Subcellular fractionation

In accordance with the manufacturer’s protocol, to determine the localization of AhR, cytoplasmic and nuclear sections were isolated and collected with Nuclear and Cytoplasmic Extraction Reagents Kit (Thermo Fisher Scientific, MA, USA).

### Nude mice and study approval

Four- to six-week-old female SCID mice were obtained from Hunan SJA Laboratory Animal Co., Ltd. (Changsha, China). Animal experiments were conducted with the approval of the Institutional Animal Care and Use Committee of Central South University of the Xiangya School of Medicine and adhered to the legal mandates and federal guidelines for animal protection and maintenance. UCHL3-overexpressing or UCHL3-silenced cells and the corresponding control cells were injected subcutaneously into the axilla of each mouse (1 × 10^7^ cells/mouse). Tumor volume and mouse weight were later measured every 3 days until sacrifice at 31 days. Tumors were weighed, fixed in 10% formalin and then paraffin embedded or used to extract RNA and protein.

### Statistical analyses

Except for the nude mouse experiments, experiments were repeated at least three times. Data are shown as the mean ± SD or SEM. GraphPad Prism 6.0 software was used to conduct statistical analyses. Student’s *t*-test was used to determine the significance of differences between two groups, and analysis of variance (ANOVA) was applied to compare more than two groups. Pearson’s correlation coefficient was used for correlation analysis. Differences were considered statistically significant in the following case: *p* < 0.05 (*p < 0.05, **p < 0.01, ***p < 0.001, ****p < 0.0001).

## Supplementary information

supplementary

supplementary marked up

## Data Availability

Not applicable.
