# Supplementary material for: The deubiquitylase UCHL3 maintains cancer stem-like properties by stabilizing the aryl hydrocarbon receptor
Source: Signal Transduct Target Ther. 2020 Jun 17;5:78. doi: 10.1038/s41392-020-0181-3 (PMC7297794; doi:10.1038/s41392-020-0181-3)
Supplement: Supplementary file 1 — supplementary [file 41392_2020_181_MOESM1_ESM.docx]

Supplementary Materials for

Deubiquitylase UCHL3 maintains cancer stem-like properties by stabilizing aryl hydrocarbon receptor

Lianlian Ouyang ^1^, Bin Yan ^2,3^, Yating Liu ^2,3^, Chao Mao ^2,3^, Min Wang ^2,3^, Na Liu ^2,3^, Zuli Wang ^2,3^, Shouping Liu^2,3^, Ying Shi ^2,3^, Ling Chen ^2,3^, Xiang Wang ^5^, Yan Cheng ^6^, Ya Cao ^2,3^, Desheng Xiao ^4^, Lingqiang Zhang^7^, Shuang Liu ^1,*^, Yongguang Tao ^2,3,5,*^,

**Affiliations:**

1. Department of Oncology, Institute of Medical Sciences, National Clinical Research Center for Geriatric Disorders, Xiangya Hospital, Central South University, Changsha, Hunan, 410008 China
2. Key Laboratory of Carcinogenesis and Cancer Invasion, Ministry of Education, Department of Pathology, Xiangya Hospital, Central South University, Hunan, 410078 China
3. Key Laboratory of Carcinogenesis of Ministry of Health, Cancer Research Institute; School of Basic Medicine, Central South University, Changsha, Hunan, 410078 China
4. Department of Pathology, Xiangya Hospital, Central South University, Changsha, Hunan, 410008 China
5. Department of Thoracic Surgery, Hunan Key Laboratory of Tumor Models and Individualized Medicine, Second Xiangya Hospital, Central South University, Changsha, China
6. Xiangya School of Pharmaceutical Sciences, Central South University, Changsha, 410008, China
7. State Key Laboratory of Proteomics, National Center for Protein Sciences (Beijing), Beijing Institute of Lifeomics, 100850, Beijing, China

* Corresponding author. S.L. Email: [shuangliu2016@csu.edu.cn](mailto:shuangliu2016@csu.edu.cn) , Department of Oncology, Xiangya Hospital, Central South University. Tel. +(86) 731-84805448; Fax. +(86) 731-84470589. Y.T. Email: [taoyong@csu.edu.cn](mailto:taoyong@csu.edu.cn), Department of Pathology, Xiangya Hospital, Central South University.

**This PDF file includes:**

Figures. S1 to S5

Tables S1 to S2

Figure. S1.

**
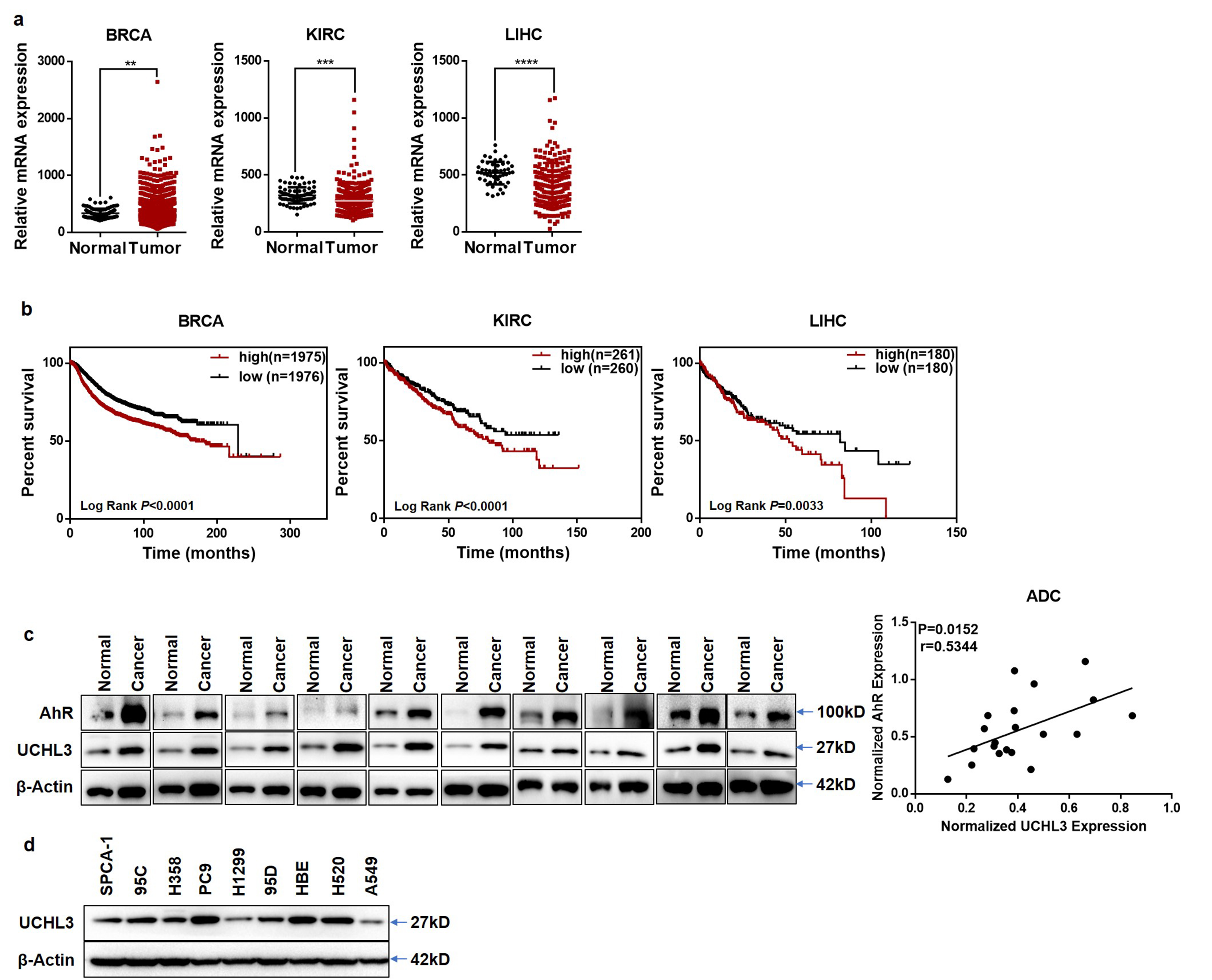
**

**Figure. S1 Detection of UCHL3 expression levels in lung cancer cell lines.**

**a** Human UCHL3 expression levels in different tumor type from TCGA (BRCA Normal n＝112 Tumor n＝1041; KIRC Normal n＝72 Tumor n＝518; LIHC Normal n＝50 Tumor n＝197). Each dot is represented as a sample. Data are shown as the mean ± SD; ** p < 0.01, *** p < 0.001, **** p < 0.0001. Tested by two-tailed Student’s t-test. **b** Overall survival rates associated with UCHL3 expression in different tumor type from TCGA. BRCA p < 0.001; KIRC p < 0.001; LIHC p＝0.0033. Tested by Log-Rank test. **c** Western blot results indicating an increased expression of UCHL3 and AhR protein levels in 10 pairs lung cancer tissues relative to adjacent normal lung tissue in ADC (n＝10). The quantification of protein strips was performed using Image J software. Protein expression level of UCHL3 and AhR is positively correlated. Statistical analyses were performed using Prism 6.0 GraphPad Software. r＝0.5344; p＝0.0152; tested by Pearson’s correlation analysis. **d** Western blot were used to detect the expression of UCHL3 in a panel of lung cancer cell lines.

Figure. S2.

**
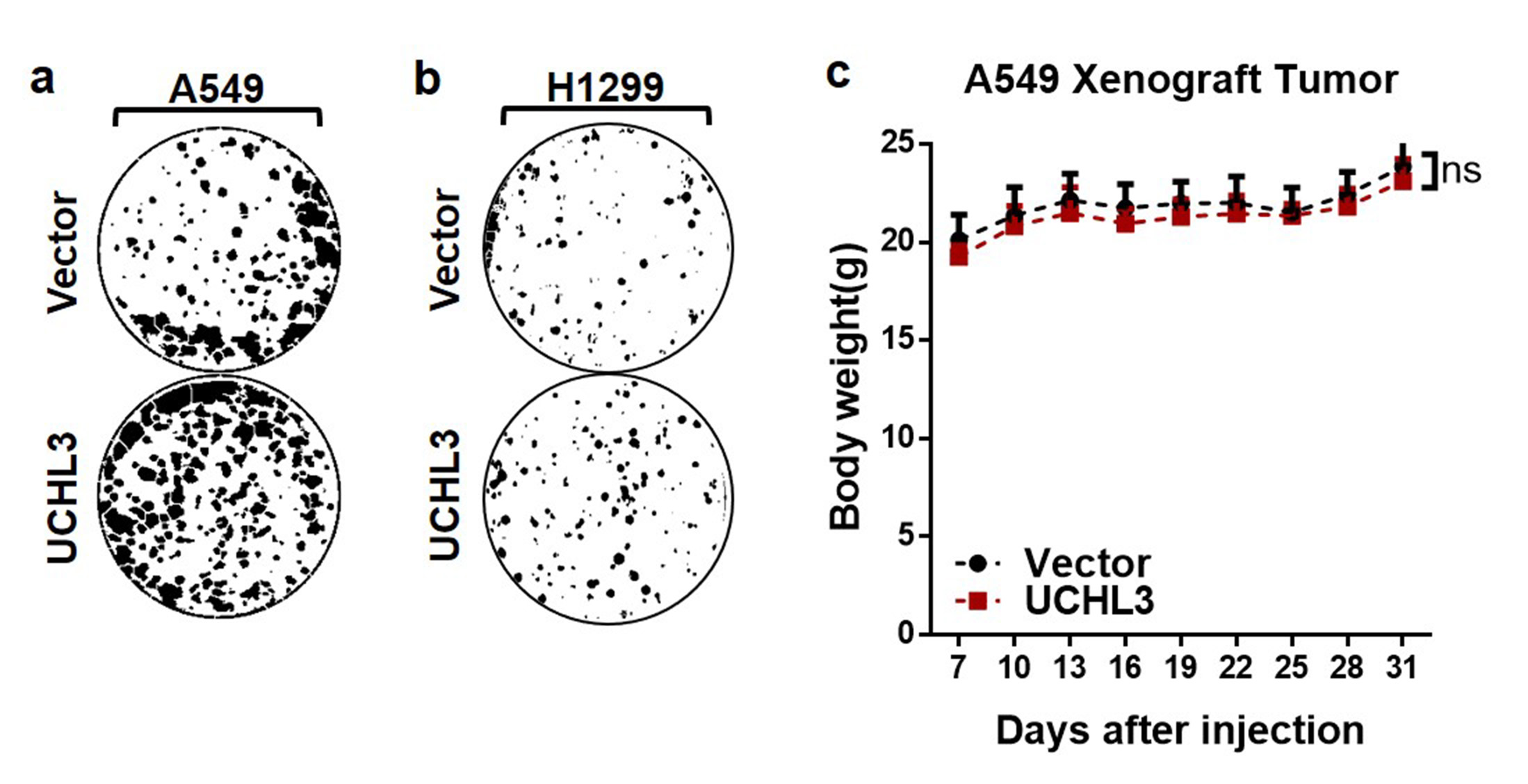
**

**Figure. S2 Images of a colony formation assay in cell lines overexpressing UCHL3 and images of nude mice xenograft tumors after the injection of cells overexpressing UCHL3.**

**a, b** Representative images of colony formation assays of A549 (a) and H1299 (b) cells which stably overexpressing UCHL3. **c** The body weight of nude mice after the injection of A549 cells which stably expressing control or UCHL3 overexpression vectors for 30 days (n＝6). Data are shown as the mean ± SD; two-tailed Student’s t-test; ns indicates nonsignificant (p ＞0.05).

Figure. S3.


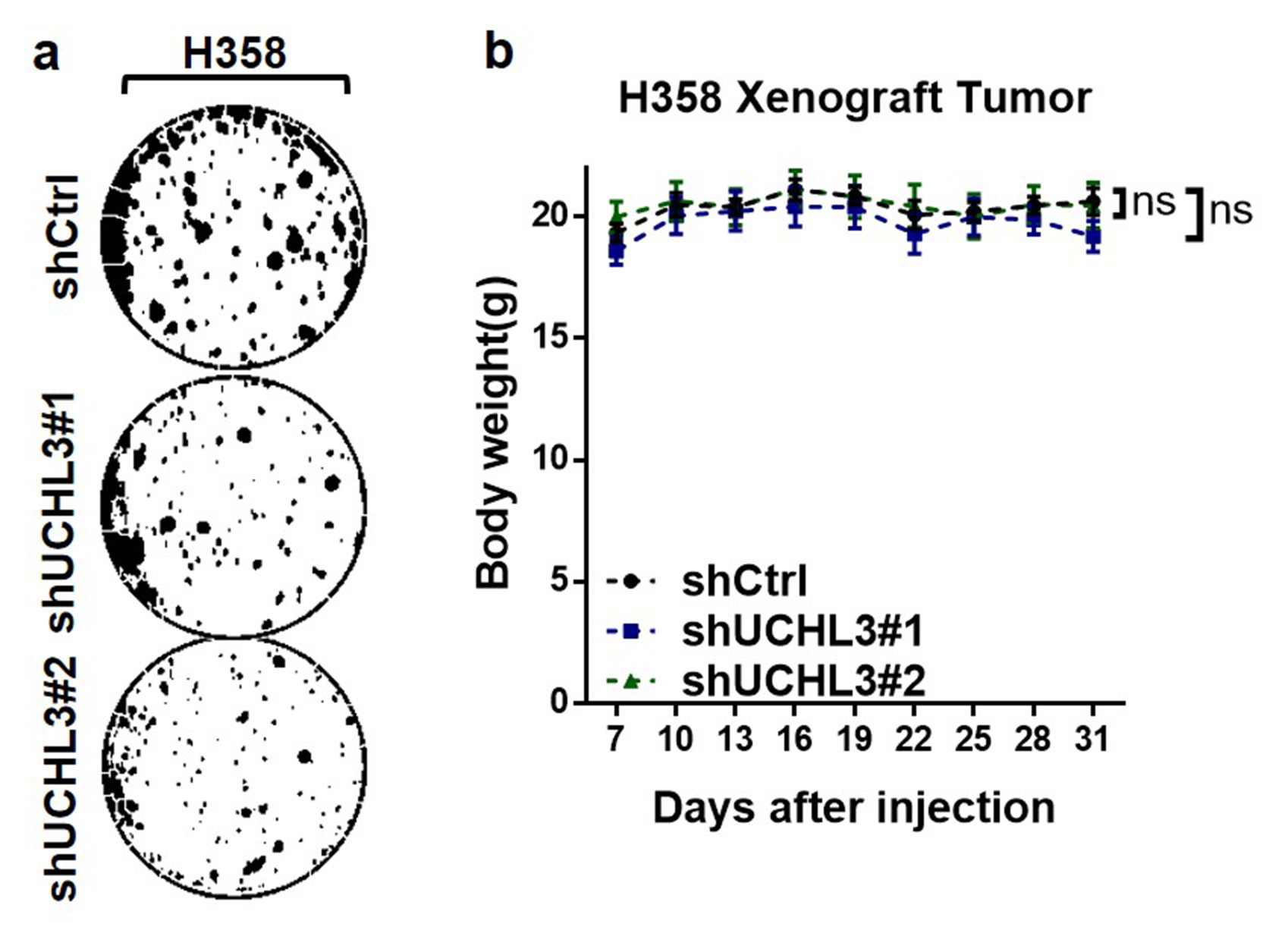


**Figure. S3** **Images of a colony formation assay in knockdown UCHL3 cell lines and images of nude mice xenograft tumor after the injection of knockdown UCHL3 cells.**

**a** Representative images of colony formation assay of H358 cells with stable knockdown of UCHL3. **b** The body weight of nude mice after the injection of H358 cells which stably expressing control or UCHL3 knockdown vectors for 30 days (n＝6). Data are shown as the mean ± SD; one-way ANOVA with multiple comparisons; ns indicates nonsignificant (p ＞0.05).

Figure. S4.


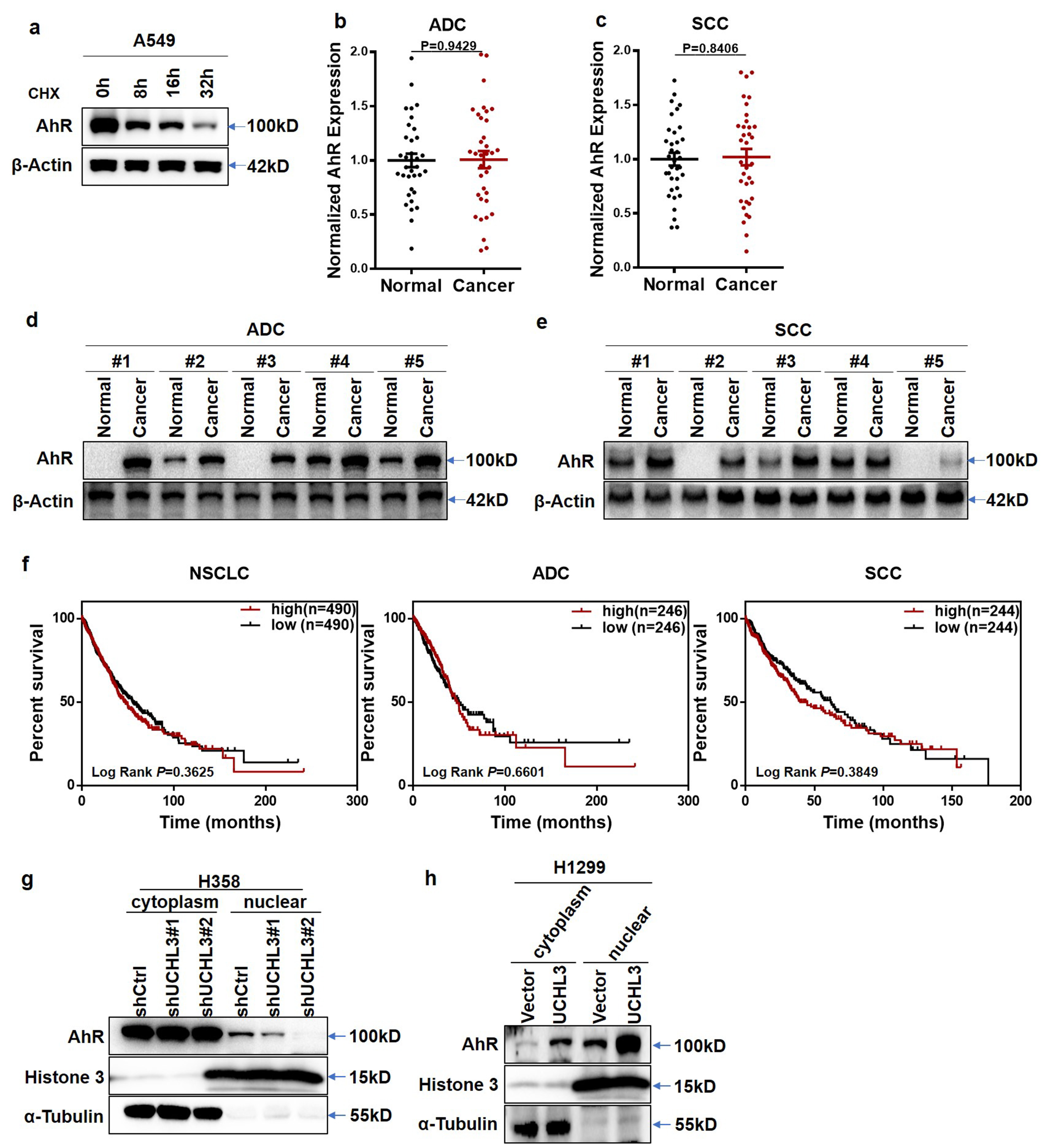


**Figure. S4 AhR protein degradation dependent on ubiquitin proteasome pathway.**

**a** A549 cells were treated with cycloheximide (CHX, 10 μg/ml) for an indicated time, and AhR protein expression was analyzed by WB. **b, c** qRT-PCR shows no difference in AhR mRNA levels in 35 paired ADC (b) and SCC (c) lung cancer samples relative to corresponding normal lung tissue samples (n＝35). Data are shown as the mean ± SD; ADC p＝0.9429; SCC p＝0.8406. Tested by two-tailed Student’s t-test. **d, e** Western blot results show increased AhR expression in 10 paired ADC (d) and SCC (e) lung cancer and corresponding normal lung-tissue samples. **f** Kaplan-Meier curves for overall survival rates associated with AhR expression in lung cancer. NSCLC p＝0.3625; ADC p＝0.6601; SCC p＝0.3849. Tested by Log-Rank test. **g h** Western blot detect the AhR level in the nuclear and cytosolic fractions derived from UCHL3 overexpression H1299 (h) and knockdown H358 (g) cells.

Figure. S5.


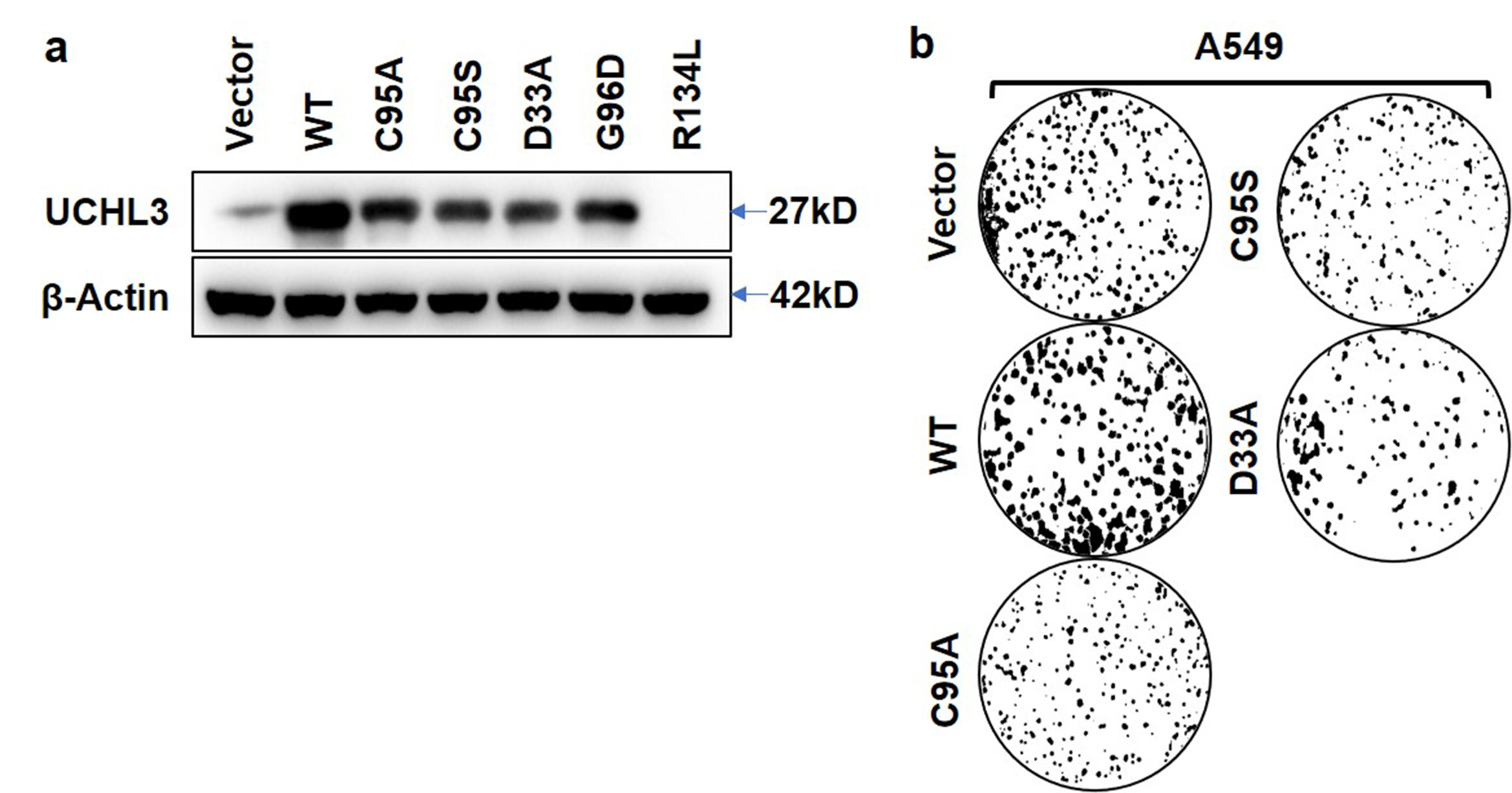


**Figure. S5 UCHL3 and UCHL3 point mutations.**

**a** The following vectors were transiently overexpressed in HEK293T cells: UCHL3, UCHL3-C95A, UCHL3-C95S, UCHL3-D33A, UCHL3-G96D and UCHL3-R134L. UCHL3 protein levels were detected by Western blot. **b** Representative images of a colony formation assays of A549 cells stably overexpressing UCHL3 and UCHL3 point mutations.

Table S1.

**Table. S1** shRNA target sequences used in these studies

| Name | Source | Target Sequences |
| --- | --- | --- |
| shUCHL3#1 | Genechem | GCACCAAGTATAGATGAG |
| shUCHL3#2 | Genechem | GTCTTACTTCTCTTTCCTA |
| shUCHL3#3 | Genechem | CCTGGAGGAATCTGTGTC |
| shUCHL3#4  shAhR#1 | Genechem  Genechem | GTCAATAATGGAAACACC  AATGATTAAGACTGGAGAA |
| shAhR#2 | Genechem | ATAATAACTCCTCAGACAT |

Table S2.

**Table. S2** Primers for ChIP enrichment detection at selected regions as indicated genes

| Name | Full Name | Gene ID | | Sequences |
| --- | --- | --- | --- | --- |
| ABCG2  c-Myc  ALDH1A1  KLF4 | ATP binding cassette subfamily G member 2  v-myc avian myelocytomatosis viral oncogene homolog  aldehyde dehydrogenase 1 family member A1  Kruppel-like factor 4 | | 9429  4609  216  9314 | F: CAAGAGCAGGCAGGAAGGA  R: GCAGAGACAGTGGTAATACGAA  F: TACGGAGGAGCAGCAGAGA  R: GGCGGAGATTAGCGAGAGAG  F: AGAACCAAATTGCTGAGCCA  R: CAAACCCGAGTCAAAGCAGA  F: GGAGAGTGCGTGGCTTGAA  R: GTCTCGAACACCTGACCTCAA |
